# The ontogeny of sleep-wake cycles in zebrafish: a comparison to humans

**DOI:** 10.3389/fncir.2013.00178

**Published:** 2013-11-13

**Authors:** Amanda Sorribes, Haraldur Þorsteinsson, Hrönn Arnardóttir, Ingibjörg Þ. Jóhannesdóttir, Benjamín Sigurgeirsson, Gonzalo G. de Polavieja, Karl Æ. Karlsson

**Affiliations:** ^1^Instituto Cajal, Consejo Superior de Investigaciones CientíficasMadrid, Spain; ^2^Biomedical Engineering, School of Science and Engineering, Reykjavik UniversityReykjavik, Iceland; ^3^3Z PharmaceuticalsReykjavik, Iceland

**Keywords:** sleep, wakefulness, *Danio rerio*, humans, ontogeny, bout structure

## Abstract

Zebrafish (*Danio rerio*) are used extensively in sleep research; both to further understanding of sleep in general and also as a model of human sleep. To date, sleep studies have been performed in larval and adult zebrafish but no efforts have been made to document the ontogeny of zebrafish sleep–wake cycles. Because sleep differs across phylogeny and ontogeny it is important to validate the use of zebrafish in elucidating the neural substrates of sleep. Here we describe the development of sleep and wake across the zebrafish lifespan and how it compares to humans. We find power-law distributions to best fit wake bout data but demonstrate that exponential distributions, previously used to describe sleep bout distributions, fail to adequately account for the data in either species. Regardless, the data reveal remarkable similarities in the ontogeny of sleep cycles in zebrafish and humans. Moreover, as seen in other organisms, zebrafish sleep levels are highest early in ontogeny and sleep and wake bouts gradually consolidate to form the adult sleep pattern. Finally, sleep percentage, bout duration, bout number, and sleep fragmentation are shown to allow for meaningful comparisons between zebrafish and human sleep.

## INTRODUCTION

In all species studied so far, sleep levels are highest and sleep bouts are shortest early in ontogeny (Kleitman and Engelman, 1951; Roffwarg et al., 1966; Jouvet-Mounier et al., 1970; Blumberg et al., 2005; Jenni et al., 2006). Cetaceans, however, represent an exception from this rule (Lyamin et al., 2005). For example, human infants spend about two-thirds of the day (i.e., 24-h) asleep whereas adults sleep for only one-third of the day (Roffwarg et al., 1966). This reduction in sleep levels across ontogeny has been reported in many other species (Jouvet-Mounier et al., 1970; Hoppenbrouwers and Sterman, 1975; McGinty et al., 1977). Another seemingly universal feature of sleep development is the gradual consolidation of sleep and wake bouts (Blumberg et al., 2005; Arnardóttir et al., 2010; Karlsson et al., 2011). For example, the average length of a sleep bout in 2-day-old rats is less than 25 s, over the next 14 days this average length increases to about 100 s (Blumberg et al., 2005) and the same trend has been demonstrated in other rodent species and humans; in flies sleep is more abundant in young rather than old flies (Shaw et al., 2000; Jenni et al., 2006; Todd et al., 2012). Even though sleep has been studied in larvae and adults, no such description of sleep–wake ontogeny exists for zebrafish (*Danio rerio*), a recent, promising model for sleep research (Zhdanova, 2006; Zhdanova et al., 2006; Yokogawa et al., 2007; Rihel et al., 2010a, b; Sigurgeirsson et al., 2011).

Zebrafish are a highly regarded model in developmental biology because of their fecundity, larval-stage transparency, short time to hatching, and ease in handling (Eisen, 1996); furthermore, they are well characterized in terms of development, neurobiology, and genetics (Westerfield, 2000). Sleep in zebrafish (adults as well as larvae) is measured in behavioral assays where it has been shown to exhibit the hallmarks of mammalian sleep, that is, (1) the absence of voluntary movement; (2) reversibility; (3) spontaneous occurrence with a circadian rhythm; (4) increased arousal thresholds; and (5) homeostatic regulation (Prober et al., 2006; Zhdanova, 2006; Zhdanova et al., 2006; Yokogawa et al., 2007). Moreover, zebrafish utilize all neurotransmitters currently known to be important for the regulation of sleep and wakefulness (Panula et al., 2010), and respond similarly to mammals when exposed to pharmacological agents that promote either sleep or wakefulness (Rihel et al., 2010a; Sigurgeirsson et al., 2011). In addition, there are many experimental tools (e.g., morpholino-oligonucleotide knock-downs, optical imaging techniques, large forward genetic screens), not easily applied in mammalian models, that can be readily applied to zebrafish (McLean and Fetcho, 2008; Appelbaum et al., 2010; Friedrich et al., 2010; Naumann et al., 2010; Bedell et al., 2012). Neural circuits driving sleep–wake cycles have only just begun to be delineated in zebrafish.

Many attempts have been made to provide a mathematical description of sleep (Achermann and Borbely, 1990, 1994, 2003; Borbely and Achermann, 1999; Best et al., 2007; Rempe et al., 2010) as well as provide a description of the sleep–wake bout dynamics (Lo et al., 2002, 2004; Blumberg et al., 2005, 2007a; Bianchi et al., 2010; Chu-Shore et al., 2010). These efforts have mostly been confined to mammalian data and our knowledge this has not been attempted for fish or invertebrates. Briefly, in adult mammals, sleep bouts have been shown to fit exponential distributions whereas wake bouts have been shown to fit power-law distributions (Lo et al., 2002, 2004; Blumberg et al., 2005; Arnardóttir et al., 2010; Karlsson et al., 2011). Quantification of sleep architecture in terms of bout duration and state transitions has important applications; e.g., state transition data has been used to monitor sleep quality in obstructive sleep apnea (Bianchi et al., 2010) and it has been used to gage developmental milestones in sleep development (Blumberg et al., 2005, 2007b). Since transitions between sleep states [i.e., rapid eye movement (REM)/non-REM (NREM) alternations] and events within a sleep state (e.g., micro-arousals, K-complexes, and apneas) are ignored, state transition analysis can be performed identically in different animals making sleep states comparable across ontogeny and phylogeny. In mammals, the waking state is largely believed to be represented by high tonic activity of brainstem neurons releasing noradrenaline, histamine, and serotonin (Pace-Schott and Hobson, 2002; McCarley, 2007). Acetylcholine is released both at the level of basal forebrain and brainstem and hypothalamic hypocretin is released in concert with arousal peaks (McCarley, 2007; Blouin et al., 2013); even though in the absence of hypocretin function both sleep and wake are de-stabilized (Saper et al., 2001). All of these wake-active neurons form a circuit that maintains arousal (including cortical arousal in mammals) and, during REM, dis-facilitate motor neurons via activation of GABA-ergic interneurons (Siegel, 2000). Hypothalamic melatonin and neural peptides are highly conserved in zebrafish (Appelbaum et al., 2009; Berman et al., 2009). The transition to sleep may depend on the activation of GABA-ergic neurons in the ventrolateral preoptic area which inhibit all the wake-active monoamine-ergic and hypocretin cells (Saper et al., 2001). These state transition dynamics likely reflect differences in the neural substrates governing sleep–wake cycles.

To substantiate the use of zebrafish in sleep research; as a model of human sleep and sleep disorders and their use in screening pharmacological sleep aids (Zon and Peterson, 2005; Rihel and Schier, 2012), we present data on the development of sleep and sleep–wake bout architecture across the zebrafish lifespan. First, we describe sleep across ontogeny in humans and zebrafish and describe its development. Next, we contrast sleep development between zebrafish and humans. We show that sleep–wake dynamics and bout structure follow similar developmental trajectories in humans and zebrafish.

## MATERIALS AND METHODS

### HUMANS

#### Participants

Fifty participants, from 2- to 74-year-olds, participated in the study. The data collection has previously been presented in detail (Arnardóttir et al., 2010) but in the current study the data are re-analyzed using different methods and age groups. Briefly, data were sampled cross-sectionally from multiple ages representative of the human lifespan. The groups were as follows: Children (ages 2–8, *n* = 15), Preteen and teens (ages 11–16, *n* = 9), Adults (ages 23–43, *n* = 15), and Adults (ages 49–74, *n* = 11), from here on referred to as “Adults 50+.” The study was approved by the Icelandic National Bioethics Committee (permit VSNb2007100011/03-15).

#### Recruitment

Participants were drawn from a randomized sample of 1000 inhabitants from the Reykjavik area, taken from the national registry of Iceland. Participants (or their legal guardians) were contacted by phone and offered to take part in the study. Of over 250 potential participants contacted, 78 accepted to take part and were pre-screened for health status over the phone; subsequently 21 participants (9 female, 12 male; age range 13–66 years) were dropped from the study before undergoing the polysomnography (PSG) due to at least one of the following conditions that may alter sleep patterns: obesity, depression, insomnia, snoring, alcohol and/or substance abuse, recent hospitalization, or the use of sleep altering medications. One participant underwent PSG but was dropped due to suspected hypothyroidism and six PSGs were unusable for technical reasons.

#### Questionnaire

Before the study, each participant (or their legal guardians) completed a 38-item questionnaire on sleep habits, adapted from the National University Hospital of Iceland and the Epworth sleepiness scale (ESS; Johns, 1991). The questionnaire included: five items about smoking and alcohol use, open ended questions on medical conditions, hospitalizations, and use of prescription medicines; 23 items on sleep quality; four items aimed at identifying complaints of restless leg syndrome, and six items aimed at identifying complaints of respiratory disturbances. None of the participants that provided data for the present study suffered from any condition that could have altered sleep patterns.

#### Procedure

Each participant underwent an unattended ambulatory PSG with a digital recording system (Medcare Inc., Iceland). The experimenter prepared the recording at the participant’s house after 21:00 and instructed the participant to follow his normal daily sleep routine as closely as possible. All recordings were made between 22:00 and 08:00. The PSG included a four-channel (C3-A2; C4-A1; O3-A2; O4-A1) electroencephalogram (EEG), electrooculogram (EOG), chin electromyogram (EMG), and electrocardiogram (ECG). Airflow was recorded via nasal cannula. Thoracic and abdominal respiratory movements were recorded with plethysmography. Arterial oxygen saturation was measured continuously via an infrared finger probe and a piezo-electric sensor was used to monitor postural changes.

#### Data preprocessing

Sleep–wake cycles were scored by an accredited sleep technologist. All sleep parameters were scored in accordance to the Rechtschaffen and Kales criteria (Rechtschaffen and Kales, 1968). In all groups sleep was scored in 30-s epochs according to conventional methods (Rechtschaffen and Kales, 1968) using the Neuroscore software (DSI). After conventional sleep–wake scoring and analysis, all bouts of SWS 1–4 and REM were merged, since subsequent analyses do not require information on alternations within sleep states (or other parameters such as respiratory indexes, micro-arousals, etc.).

### ZEBRAFISH

#### Fish

Stock fish of the Tübingen strain were provided by the University of Oregon Zebrafish International Resource Center. Fish were fed twice a day on a diet of TetraMin flakes (Tetra Holding GmbH) and kept in a 14:10 light:dark cycle (lights-on at 07:00) in either a 3- or 10-L multi-tank constant flow system tanks (Aquatic Habitats). Water temperature was held at a constant 28.5°C, and water was replaced at a rate of 10% per day. Zebrafish eggs were harvested between 08:00 and 10:00 and placed in a separate tank (with methylene blue) until hatching. All procedures were in compliance with the regulations of the National Bioethics Committee of Iceland; permit issued to Karl Æ. Karlsson, May 19, 2008 (no number).

#### Procedure

Sixty-one zebrafish were assigned to one of four age groups: 6–10 days post-fertilization (dpf; *n* = 16); 4–6 weeks (*n* = 16); 4–6 months (*n* = 14); over 12 months (*n* = 15). These age groups roughly represent larval, juvenile, adult, and senior zebrafish, respectively. It is important to note that while there are four age groups assigned for both fish and humans, there is no experimental data available that allows them to be fully equated. Regardless, analysis of sleep behavior for groups 1–2 occurred in 24- and 12-well plates, respectively, while groups 3–4 were studied in 75-L aquarium (at 28.5°C), with a black divider setup keeping fish isolated within an environment of 10 cm across, 6.5 cm deep, and 13 cm tall. All data were collected using Ethovision XT 7.0 behavioral tracking system (Noldus Information Technology) under white and IR lights for 48 h. All groups were recorded under the same temperature (28.5°C) and 14–10 light cycle with lights-on at 07:00, lights off at 21:00. The recording commenced at 12:00; data following 24-h acclimation period was used for analysis. During recording and acclimation larvae and adult fish were fed daily at 12:00, zebrafish larval food (Zeigler Bros) and TetraMin flakes (Tetra Holding GmbH), respectively. Feeding was done at 12:00 noon. No recording was made over the seconds that took to dispense the food. Each recording was 24 h; reset at 12:00 (the food items are too small to be tracked; minimal tracking size was set at 25 pixels). No special care was taken to avoid monitoring the movements following feeding.

Larval and young zebrafish (two youngest groups) were placed individually into wells and plates were then placed in a custom-built transparent Plexiglas holder with circulating water; the holder was placed in the activity monitoring system, which was blocked from daylight and illuminated from below with white (255 lx; light-phase) or infrared light (0 lx; dark-phase). The velocity of each fish was tracked in two dimensions, at 8.33 Hz using a Sony XC-E150 infrared camera (Sony Inc.) with a 50-mm CCTV Pentax lens (Pentax, GMBH).

Young adult and adult zebrafish (two oldest groups) were placed in the recording tank at 12:00. The aquarium was illuminated with two infrared lights (0 lx; dark-phase) and a fluorescent light (255 lx; light-phase), all of which was housed in an opaque black plexiglas box with an ambient lighting of 0 lx. All data tracking and recording is identical to what is described for larvae.

#### Data preprocessing

Behavioral states were dichotomized into 1-s bins of movement/non-movement. Prior, in-depth frame-by-frame video analysis by three independent raters resulted in the adoption of the speed of 0.5 cm/s as the threshold for movement for larval zebrafish (Sigurgeirsson et al., 2011). All activity that was slower than that threshold was described as non-movement. Due to the changes in the size of the fish the threshold for movement had to be adjusted for age. A comparison between a threshold determined by mathematical scaling with body size and a threshold obtained by visual video analysis did not suggest a significant difference between these two approaches. The thresholds for groups 2–4 were set as follows: group 2: 0.75 cm/s; group 3: 1.0 cm/s; group 4: 1.5 cm/s. Following previously established criteria in adult zebrafish (Yokogawa et al., 2007; Zhdanova et al., 2008; Singh et al., 2013) after six or more consecutive 1-s bins of non-movement, the seventh second and above were classified as sleep; all other bouts were classified as wake. In prior work we applied the same criteria to larval fish (Sigurgeirsson et al., 2011). We validated this approach by calculating the response time to a 60-s light stimulus (550 lx) in 252 additional 6-dpf larvae as a function of immobile time during night. Three categories of immobile time were compared: 0–6 s, 6–11 s, and 11 and above. Mean response times were 2.28, 6.66, and 6.67 s, respectively. 0–6 s group differed from 6–11 s group (*t* = -2.256, df = 25.57, *p* = 0.033) and from 11+ group (*t* = 2.33, df = 15.74, *p* = 0.41). The 6–11 and 11+ group did not differ (*t* = 0.002, df = 34, *p* = 0.998).

### DATA ANALYSIS

#### Statistical analysis

All sleep–wake bout data were imported into Matlab 2010a (The MathWorks Inc.) for subsequent data analysis. For each individual, mean durations of sleep and wake bouts were calculated and fragmentation indexes were calculated as the number of sleep (wake) bouts divided by the total sleep (wake) time, in minutes. Analysis of variance (ANOVA) was used to test for the influence of age on percent sleep/wake, mean durations, number of bouts and fragmentation indexes, while Holm–Bonferroni corrected multiple two-tailed *t*-tests were used to test the specific differences between the age groups, with the family-wise type I error rate (alpha) set to 0.05. One of three different implementations of the *t*-test was applied for each comparison, depending on the characteristics of the two test samples. If both sets were normally distributed (or rather, failed to be rejected as coming from a normal distribution by the Lilliefors test of normality) and the variances were not unequal (i.e., “equal,” as determined by a two-sample *F*-test for equal variances) the standard parametric Student’s *t*-test was used. If the variances were unequal, the Welch’s *t*-test was used. Finally, if at least one of the two samples were not normally distributed the *p*-value was instead estimated by first bootstrapping the *t*-statistic, with a resampling of *n* = 10,000 and then calculating the probability of finding a result at least as extreme as the test *t*-statistic.

Group results are presented as mean ± standard error of the mean. In the box plots, the whiskers extend to the lowest and highest values within the 1.5 interquartile range (IQR). This corresponds to approximately ±2.7σ and 99.3% coverage if the data are normally distributed. Values outside the 1.5 IQR are considered outliers.

#### Bout distributions

The sleep and wake bout duration distributions were tested against four models: (i) exponential distribution, *f*(*x*; τ) = 1/τ exp(-*x*/τ), (ii) stretched exponential distribution, *f*(*x*; *k*, λ) = (*k*/λ)(*x*/λ)^k-1^ exp[-(*x*/λ)^k^], (iii) power-law distribution *f*(*x*; α, *x*_low_) = (α - 1)*x*_low_^α-1^*x*^α^, and (iv) lognormal distribution *f*(*x*; μ, σ) = (1/*x*)(σ^2^2π)^-1/2^ exp[-(ln *x* - π)^2^/2σ^2^]. For all the distributions *x* = *t* - *t*_min_, where *t*_min_ is the shortest bout duration for each behavioral state as determined by the experimental procedure (30 s for human sleep and wake bouts, 6 and 1 s, respectively for zebrafish sleep and wake). The exponential distribution, power-law and lognormal distribution parameters were estimated using maximum likelihood estimators (MLE). To find the power-law best fit estimates for the exponent α and lower cut-off *x*_low_ the method described in Clauset et al. (2009) was used. To estimate the best fit parameters for the stretched exponential distribution the linear fit between log(*x*) and log[-log(*y*)], where *y* is the survival distribution was used, as it has previously been shown to robustly estimate the stretched exponential parameters for small sample sizes (Sorribes et al., 2011).

## RESULTS

### HUMAN SLEEP RATIO DECREASES WITH AGE

**FIGURE 1:**
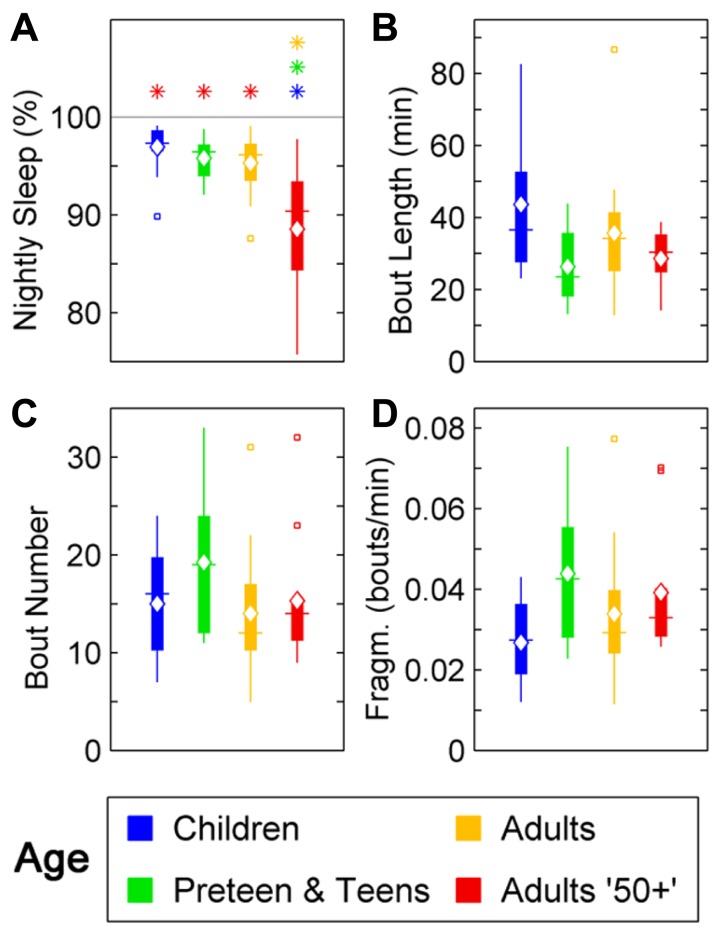
**Sleep parameters during the night in humans across age groups: Children (blue), Preteens and teens (green), Adults (orange), and Adults 50 + (red).** Colored asterisks above bars indicate a statistically significant difference between the bar group and the group with the corresponding color of the asterisk. **(A)** Percentage sleep time. The mean percentage of sleep is significantly lower in Adults older than 50 than in all other age groups. **(B)** Mean sleep bout length. There is no difference between age groups in the mean duration of the sleep bouts. **(C)** The number of sleep bouts during nightly sleep do not change across the age groups. **(D)** Sleep bout fragmentation (bouts/min). There are no significant changes of the sleep fragmentation with age. Diamond, white line, and squares indicate mean, median, and outlier values, respectively.

In humans, a reduction in sleep ratio (percentage of sleep across the night) was found across age [*F*(3,46) = 11,69, *p* < 0.0001]. The Adults 50+ age group had a lower sleep ratio compared to Children (*p* < 0.01), Preteens and teens (*p* < 0.01), and Adults (*p* < 0.01; **Figure 1A**; **Table 1**). No changes were found in the average length of sleep bouts, number of sleep bouts or sleep fragmentation across age (**Figures 1B–D**; **Table 1**).

**Table 1:** Sleep and wake parameters across ontogeny in humans and zebrafish.

	Age group	Percent time of night (%)		Bout length (min)		Total number of bouts		Fragmentation (bouts/min)		Number of different bout lengths		Favored model	Model shape parameter	
		Mean	SE	Mean	SE	Mean	SE	Mean	SE	Mean	SE		Mean ± SE
Human sleep	Children	96.96	0.63	43.64	4.89	15.00	1.46	0.03	0.00	13.60	1.14	Stretched exponential	κ = 0.84 ± 0.06
	Preteen and teens	95.82	0.71	26.33	3.48	19.22	2.71	0.04	0.01	16.56	2.01	Stretched exponential	κ = 0.84 ± 0.05
	Adults	95.32	0.80	35.62	4.44	14.00	1.67	0.03	0.00	12.47	1.20	Stretched exponential	κ = 0.79 ± 0.07
	Adults ‘50+’	88.54	1.93	28.62	2.58	15.27	2.01	0.04	0.00	13.64	1.34	Power-law	α = 2.63 ± 0.48
Zebrafish sleep	6–10 days	58.17	4.25	0.44	0.15	1315.69	135.74	4.49	0.59	94.69	8.10	Stretched exponential	κ = 0.69 ± 0.03
	4–6 weeks	54.25	5.02	0.32	0.05	1144.44	63.22	4.52	0.75	94.31	8.72	Stretched exponential	κ = 0.77 ± 0.03
	4–6 months	33.37	4.40	0.30	0.04	674.86	62.11	4.37	0.85	81.93	7.20	Stretched exponential	κ = 0.71 ± 0.02
	12 months+	33.06	6.44	0.41	0.11	506.40	79.13	5.43	1.29	75.60	11.20	Power-law	α = 2.80 ± 0.17
Human wake	Children	3.04	0.63	1.21	0.21	14.07	1.43	1.03	0.10	3.93	0.36	Power-law	α = 1.78 ± 0.04
	Preteen and teens	4.18	0.71	1.16	0.30	18.22	2.71	1.09	0.13	4.22	0.40	Power-law	α = 1.90 ± 0.04
	Adults	4.68	0.80	1.61	0.22	13.00	1.67	0.81	0.12	4.53	0.48	Power-law	α = 1.71 ± 0.03
	Adults ‘50+’	11.46	1.93	4.01	0.85	14.36	1.98	0.40	0.09	6.36	0.74	Power-law	α = 1.83 ± 0.13
Zebrafish wake	6–10 days	41.83	4.25	0.19	0.01	1315.63	135.68	5.56	0.26	42.81	4.07	Power-law	α = 4.09 ± 0.44
	4–6 weeks	45.75	5.02	0.23	0.02	1144.44	63.22	4.64	0.30	51.75	5.35	Power-law	α = 3.96 ± 0.11
	4–6 months	66.63	4.40	0.77	0.18	674.00	62.11	1.94	0.27	85.93	5.55	Power-law	α = 2.57 ± 0.11
	12 months+	66.94	6.44	1.52	0.54	505.67	79.17	1.65	0.31	85.27	7.91	Power-law	α = 2.45 ± 0.13

### HUMAN AWAKENINGS AT NIGHT LENGTHEN WITH AGE

**FIGURE 2:**
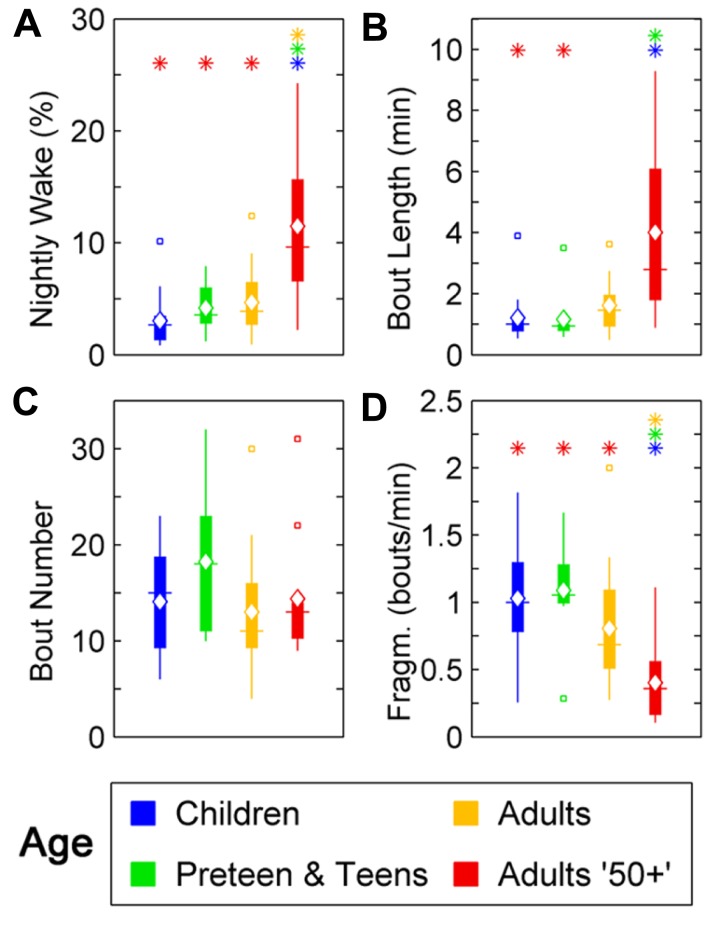
**Wake parameters of nightly sleep in humans across age groups: Children (blue), Preteens and teens (green), Adults (orange), and Adults 50 + (red).** Colored asterisks above bars indicate a statistically significant difference between the bar group and the group with the corresponding color of the asterisk. **(A)** Percentage wake time. The mean percentage of time awake is significantly lower for Adults over 50 than for all other age groups. **(B)** Mean wake bout duration. Adults over 50 have significantly longer wake bouts during the night than Children and Preteens and teens. **(C)** The number of wake bouts do not change with age. **(D)** Wake bout fragmentation (bouts/min). Adults over 50 show significantly less fragmented wake than the younger age groups. Diamond, white line, and squares indicate mean, median, and outlier values, respectively.

Changes were seen in wake ratio (percentage of time spent awake during night) [*F*(3,46) = 11.69, *p* < 0.0001]. Wake percentage increased significantly after the age of 50 compared to Children (*p* < 0.01), Preteens and teens (*p* < 0.01), and Adults (*p* < 0.01; **Figure 2A**; **Table 1**). Average wake bout length also increased with age [*F*(3,46) = 9.08, *p* < 0.0001]. Wake bouts were longer in the age group Adults 50+ compared to Children (*p* < 0.0001) and Preteens and teens (*p* < 0.0001; **Figure 2B**; **Table 1**). However, wake bout number was unchanged across age (**Figure 2C**; **Table 1**). Wake fragmentation, defined as the number of awakenings divided by the total time awake, decreased after the age of 50 [*F*(3,46) = 6.94, *p* < 0.001; **Figure 2D**; **Table 1**], that is, the Adults 50+ age group showed reduced wake fragmentation (i.e., when awake, the wake bout is longer) compared to Children (*p* < 0.0001), Preteens and teens (*p* < 0.001), and Adults (*p* < 0.0001).

### ZEBRAFISH SLEEP RATIO DECREASES WITH AGE AND SLEEP BOUTS CONSOLIDATE

**FIGURE 3:**
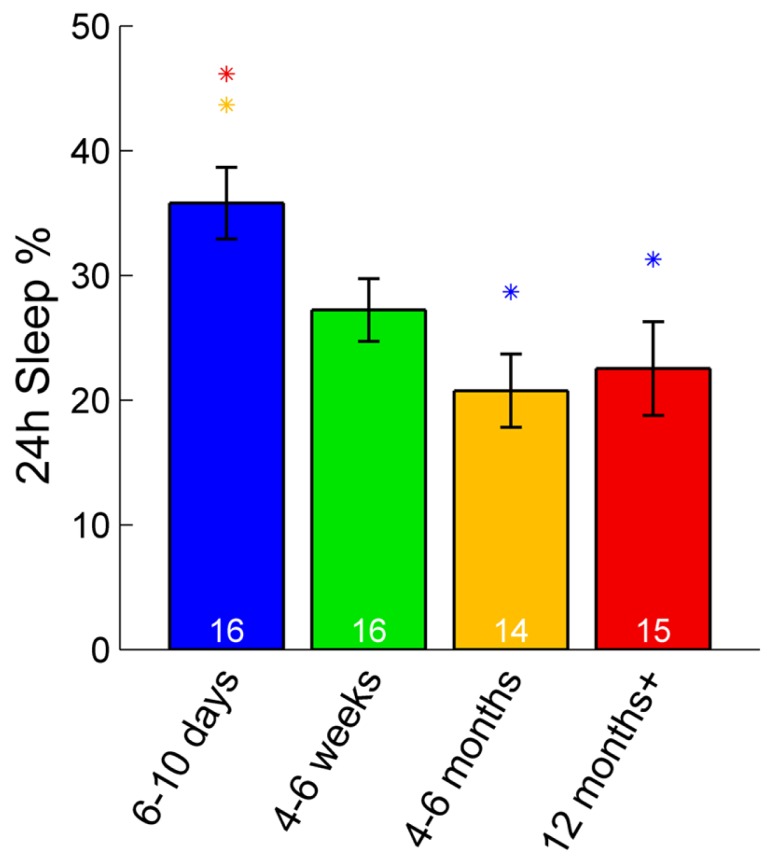
**Sleep percentage across 24 h in zebrafish.** The youngest group (6–10 days post-fertilization) sleep significantly more over a full 24-h time period than adult fish (4–6 and 12+ months). Colored asterisks above bars indicate a statistically significant difference between the bar group and the group with the corresponding color of the asterisk. White numbers on the bars indicate the number of zebrafish in each age group.

**FIGURE 4:**
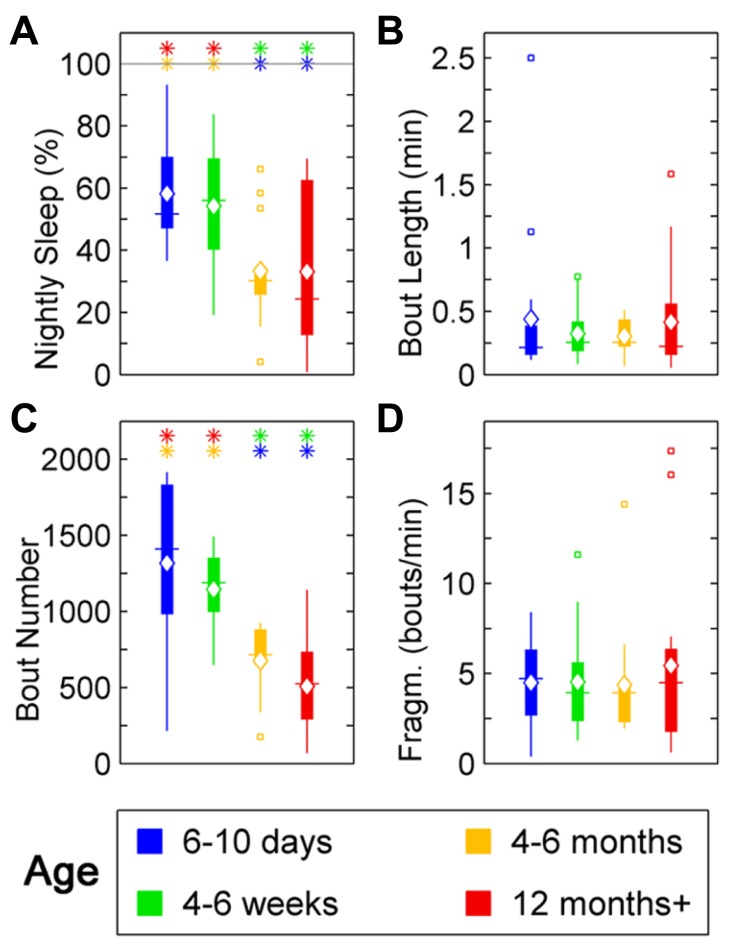
**Sleep parameters for zebrafish during the night for age groups: 4–6 days post-fertilization (group 1, blue), 4–6 weeks (group 2, green), 4–6 months (group 3, orange), and 12 months + (group 4, red).** Colored asterisks above bars indicate a statistically significant difference between the bar group and the group with the corresponding color of the asterisk. **(A)** Percentage sleep time. Larvae (groups 1 and 2) sleep significantly more than adult fish (groups 3 and 4) during the dark-phase. **(B)** There is no significant change in the mean sleep bout duration between the age groups. **(C)** Number of sleep bouts. Larvae (groups 1 and 2) show significantly more sleep–wake transitions than adult fish (groups 3 and 4). **(D)** Sleep bout fragmentation (bouts/min). No changes of the fragmentation of sleep are observed across the age groups. Diamond, white line, and squares indicate mean, median, and outlier values, respectively.

In zebrafish, sleep ratio at night decreased with age [percentage of time spent asleep during night; *F*(3,57) = 6.87, *p* < 0.001] as well as the full 24 h sleep ratio (**Figure 3**; **Table 1**). Four- to six-month-olds showed significantly decreased sleep ratio compared to the 6- to 10-day-olds (*p* < 0.001) and 4- to 6-week-olds (*p* < 0.01). The 12 month+ group also had decreased sleep ratio compared to the 6- to 10-day-old group (*p* < 0.01) and 4- to 6-week-old group (*p* < 0.05). Furthermore, in zebrafish the sleep bout number during night decreased with age [*F*(3,57) = 17.25, *p* < 0.0001]. The 4- to 6-month-old group showed significantly less number of sleep bouts compared to 6–10 days (*p* < 0.001) and 4–6 weeks old (*p* < 0.0001; **Figures 4A–C**; **Table 1**). Also the 12+ month group had fewer sleep bouts compared to 6- to 10-day-olds (*p* < 0.0001) and 4- to 6-week-old fish (*p* < 0.0001). No changes were seen across age in sleep bout length or sleep fragmentation in zebrafish (**Figures 4B–D**; **Table 1**).

### ZEBRAFISH WAKE RATIO INCREASES AND AWAKENINGS AT NIGHT LENGTHEN WITH AGE

**FIGURE 5:**
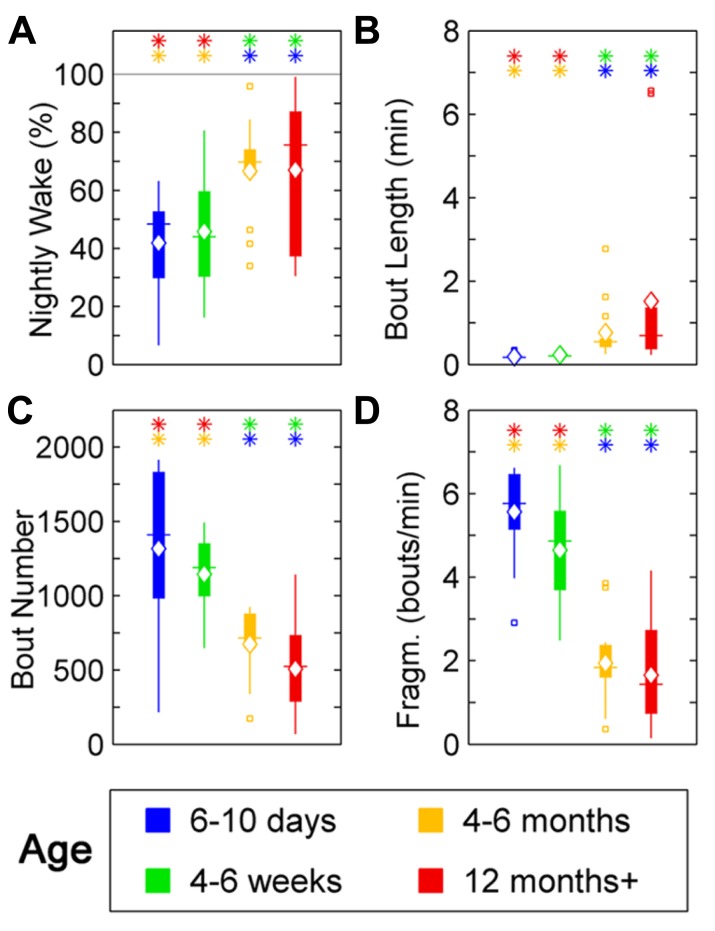
**Wake parameters for zebrafish during the night for age groups: 4–6 days post-fertilization (group 1, blue), 4–6 weeks (group 2, green), 4–6 months (group 3, orange), and 12 months + (group 4, red).** Colored asterisks above bars indicate a statistically significant difference between the bar group and the group with the corresponding color of the asterisk. **(A)** Percentage wake time. Larvae (groups 1 and 2) spend less time awake during the night than adult fish (groups 3 and 4). **(B)** Mean wake bout duration. The wake bout lengths in larvae (groups 1 and 2) are significantly shorter than in adult fish (groups 3 and 4). **(C)** Number of sleep bouts for groups 1–4. Larvae (groups 1 and 2) show significantly more sleep–wake transitions than adult fish (groups 3 and 4). **(D)** Sleep bout fragmentation (bouts/min). The wake time of larvae (groups 1 and 2) is significantly more fragmented than for adult zebrafish (groups 3 and 4). Diamond, white line, and squares indicate mean, median, and outlier values, respectively.

Wake behavior during night in zebrafish showed a clear change with age. The wake ratio increased significantly [*F*(3,57) = 6.87, *p* < 0.001], from 6- to 10-day-olds to the 12+ month old group. Specifically, 12+ month olds showed increased wake ratio compared to 6- to 10-day (*p* < 0.01) and 4- to 6-week-olds (*p* < 0.05), and the 4- to 6-month-old group had a higher wake ratio than 6- to 10-day (*p* < 0.001) and 4- to 6-week-old fish (*p* < 0.01; **Figure 5A**; **Table 1**). Wake bout length also increased with age [*F*(3,57) = 5.05, *p* < 0.01], i.e., the 12+ month old group showed increased wake bout length compared to 6- to 10-day (*p* < 0.0001) and 4- to 6-week-old group (*p* < 0.0001), and the 4–6 months group had longer wake bouts than 6–10 days (*p* < 0.0001) and 4–6 weeks old (*p* < 0.0001). Furthermore, wake bout number showed a decrease with age [*F*(3,57) = 17.29, *p* < 0.0001], i.e., the 12+ month old group had less wake bouts than 6–10 days (*p* < 0.0001) and 4–6 weeks old (*p* < 0.0001), and the 4–6 months group had fewer wake bouts than 6–10 days (*p* < 0.001) and 4- to 6-week group (*p* < 0.0001). Finally wake fragmentation decreased with age [*F*(3,57) = 45.8, *p* < 0.0001], i.e., the 12+ months group showed less fragmentation compared to 6- to 10-day (*p* < 0.0001) and 4- to 6-week-old fish (*p* < 0.0001), and the 4- to 6-month group exhibited less fragmentation compared to 6–10 days (*p* < 0.0001) and 4- to 6-week-old (*p* < 0.0001; see **Figures 5B–D**; **Table 1**).

### SLEEP BOUTS EXHIBIT A STRETCHED EXPONENTIAL BEHAVIOR AND WAKE BOUTS POWER-LAW BEHAVIOR IN BOTH HUMANS AND ZEBRAFISH

Sleep and wake bout length distributions were tested against the exponential and power-law distributions, since these have previously been found to describe human sleep–wake behavior (Lo et al., 2002, 2004; Arnardóttir et al., 2010; Bianchi et al., 2010). In addition, the stretched exponential (Weibull) distribution was included as it has been shown to describe wake dynamics in fruit flies (Sorribes et al., 2011) and the lognormal distribution as another possible full range alternative to the power-law. These distributions represent four different basic generating mechanisms commonly found throughout nature, and in particular, the observation of an exponential survival distribution implies that the state transitions are random events while a power-law or a lognormal distribution are indicative of bursty dynamics. The stretched exponential distribution, on the other hand, allows for a sliding measure between random dynamics and bursts, quantified by the shape parameter *k*, where *k* = 1 in the case of random dynamics and *k* < 1 when the state transitions appear in bursts.

The model selection procedure was followed from Clauset et al. (2009), which, for each model, briefly consisted of (i) find the best fit to the data and its corresponding Kolmogorov–Smirnov (KS) distance, (ii) draw a large number (*N*_rep_ = 10,000) of random samples from the model distribution using the estimated parameters from the data, where each random sample is the same size as the data, and (iii) perform a “plausibility” or consistency test by comparing the empirical KS distance to the ones from the randomly sampled data, yielding a *p*-value. Lastly, the Akaike Information Criterion with a correction for finite sample sizes (AICc) and the Bayesian Information Criterion (BIC) are used to select between the different plausible models.

In humans, however, sleep is characterized by relatively few sleep–wake transitions. To accurately assess the possible functional forms of the distributions of sleep and wake bouts, the numbers of unique bout durations become important, as these determine the number of points in the survival distributions. The number of different sleep bout lengths (which determines the survival distribution) were on average 13.6 (range 7–21) for Children, 16.6 (range 10–28) for Preteens and teens, 12.5 (range 5–21) for Adults, and 13.6 (range 9–25) for Adults 50+. The number of unique wake bouts lengths were even lower since most awakenings are short (and thus fall in the 30- or 60-s bins, see Materials and Methods, Human Data Preprocessing), with an average of 3.9 (range 2–7) for Children, 4.2 (range 2–6) for Preteens and teens, 4.5 (range 1–8) for Adults, and 6.4 (range 3–11) for the Adults 50+ group. Fits were performed on an individual basis, however, a minimum number of five unique bouts were considered necessary for obtaining meaningful fits. This left only five Children, four Preteens and teens, eight Adults, and eight Adults 50+, so pooled values for each group were analyzed as well for model selection.

**FIGURE 6:**
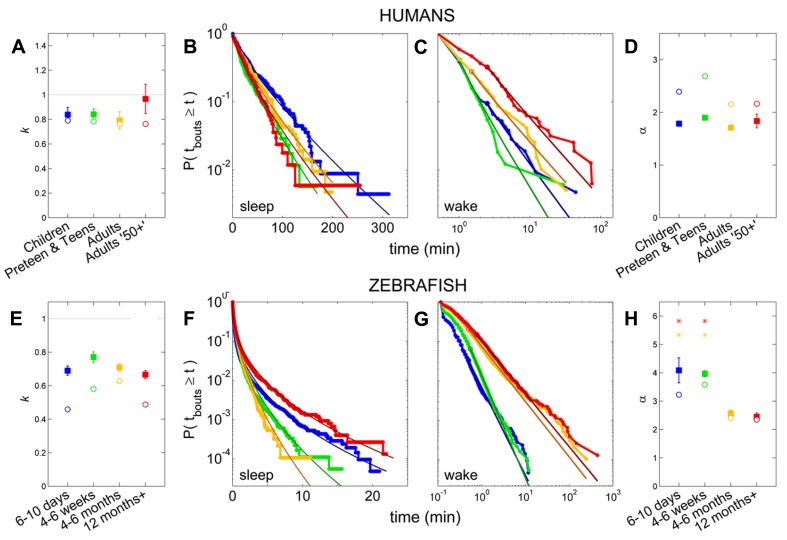
**Survivor plots of sleep and wake bouts in humans and zebrafish.** **(A–D)** Sleep and wake bout distributions and fits in humans, with Children (blue), Preteens and teens (green), Adults (orange), and Adults 50+ (red). **(E–H)** Sleep and wake bout distributions and fits in zebrafish, with 4–6 days (blue), 4–6 weeks (green), 4–6 months (orange), and 12 months + (red). **(B,F)** Sleep bout distribution of pooled values for each group, with overlain stretched exponential fits in darker shades, shown on a log-lin scale. A straight diagonal line on these axes would indicate an exponential distribution. **(A,E)** Open circles indicate the value of the shape parameter *k* of the stretched exponential fit to the pooled distributions, while filled squares show the mean of the *k*’s from fits to each individual and error bars show the standard error of the mean. The gray horizontal line marks *k* = 1, which corresponds to the special case where the stretched exponential “collapses” and becomes equal to the standard exponential distribution. No statistically significant differences between the age groups are present in either humans or zebrafish. **(C,G)** Wake bout distribution of pooled values for each group, with overlain power-law fits in darker shades, shown on a log–log scale. **(D,H)** Open circles indicate the value of the power-law exponent α from the fits to the pooled distributions, while filled squares show the mean of the α’s from the individual fits and the error bars show the standard error. For humans, no significant change is seen on the α across age. In zebrafish, the larval stages (blue, green) have statistically significantly larger power-law exponents than the adult fish (orange, red).

Pooled sleep bout distributions were found to be consistent with a stretched exponential distribution for Children (*k* = 0.79, λ = 31.6 min), Preteens and teens (*k* = 0.78, λ = 19.6 min), and the Adults group (*k* = 0.76, λ = 23.0 min), **Figures 6A,B**, whereas a power-law distribution was favored for sleep of Adults 50+ group (α = 2.2, *x*_low_ = 2.5 min). From the fits to each individual survival distribution we obtained *k* = 0.84 ± 0.06 for Children, *k* = 0.84 ± 0.05 for Preteens and teens, and *k* = 0.79 ± 0.07 for Adults (**Figure 6A**; **Table 1**). The pooled wake bout distributions were found to be well fit by a power-law for all age groups, with the exponent for Children estimated at α = 2.4 (*x*_low_ = 2.5 min), Preteens and teens α = 2.7 (*x*_low_ = 1 min), Adults α = 2.2 (*x*_low_ = 1.5 min), and Adults 50+ α = 2.2 (*x*_low_ = 2.5 min), **Figures 6C,D**. The group mean exponents were α = 1.78 ± 0.04 for Children, α = 1.90 ± 0.04 for Preteen and teens, α = 1.71 ± 0.03 for Adults, and α = 1.83 ± 0.13 for Adults 50+ (**Figure 6D**; **Table 1**). No statistically significant differences were found for neither sleep nor wake distribution parameters between age groups.

Zebrafish sleep–wake dynamics are characterized by many more transitions during the dark-phase as compared to human nocturnal sleep dynamics, on the order of 10–100 times as often (cf. **Figures 1D and 4D**). The average number of discrete sleep bouts were for the 6–10 days group 94.7 (range 58–164), 4- to 6-week group 94.3 (range 27–154), 4–6 months group 81.9 (range 22–131), and 12+ months group 75.6 (range 11–134). Similarly to human sleep–wake dynamics, we observed fewer numbers of unique wake bout durations with averages of 42.8 (range 23–88) for the 6- to 10-day-olds, 51.8 (range 23–98) for the 4–6 weeks group, 85.9 (range 56–128) for the 4–6 months and 85.3 (range 39–130) for the fish older than 12 months.

**FIGURE 7:**
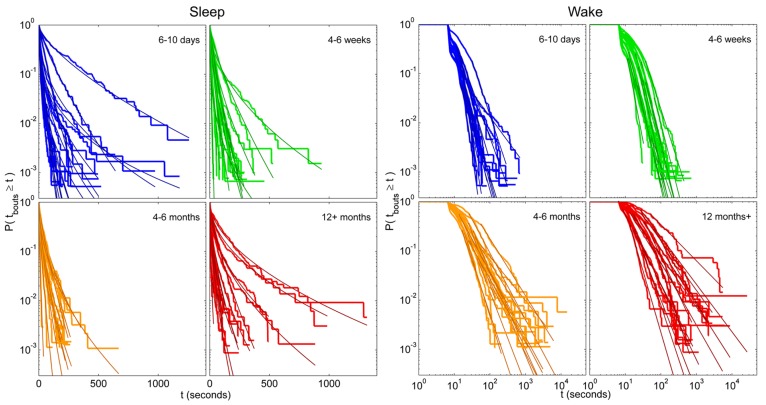
**Individual survival distributions of zebrafish sleep and wake bout lengths with corresponding stretched exponential and power-law fits.** Survivor plots of (left panel) sleep and (right panel) wake bouts in zebrafish for age groups 6–10 days (blue), 4–6 weeks (green), 4–6 months (orange), and 12 months+ (red). Each trace encompasses data from a single fish. On the left panel the axes are shown using semi-log coordinates and stretched exponential fits are overlain each individual distribution in a darker shade. Straight diagonal lines on these plots indicate an exponential distribution of bout lengths. On the right the axes are shown in log–log coordinates and power-law fits are overlain each individual distribution in a darker shade. Straight diagonal lines on these plots indicate a power-law distribution.

For each zebrafish, the exponential, stretched exponential, power-law and lognormal distributions were fit to the sleep and wake bout length distributions. The fitting procedure was performed as described above in steps i–iii, culminating in a *p*-value for each model that measures how consistent, or plausible, the model is given the data. Ideally, several models would pass as plausible for each distribution, and the AICc or BIC is then used to determine the best model. Since we are interested in assessing the effect of age on the sleep and wake distributions, we would then determine which model is most frequently found to be a good fit for the individuals of each age, and so compare the age groups. For the empirical sleep bout data, however, only 54% (33 of 61) of the fits were found to be consistent with one of the models, and of these, 79% were only consistent with a single model. For the wake bout distributions we found a similar situation, with 36% consistent with one or more models, of which 91% were consistent with only one. Since AIC and BIC should be used to compare between plausible models and in the vast majority of cases there was only one in question, we instead quantified the most probable model out of the four for each age group. These results may seem like low “hit rates” and one may be tempted to try fitting more distributions with more complicated expressions, but the downside to that approach is that the introduction of more parameters makes it difficult to interpret the distribution parameters and what they say about the sleep–wake dynamics. Another reason for not delving into a large number of test models is that many of the individual distributions are noisy, and are therefore very unlikely to ever be consistent with the still reasonably simple model distributions that we would likely test. This is probably most easily seen visually (see **Figure 7**) where we see that the fits seem quite good, despite the noisy data and often failed plausibility tests. It is important to note here, that a failed plausibility test only indicates that the data does not purely follow the exact functional form of the model, and does not rule out that the model could still be useful as a tool for measuring and comparing different distributions that show a reasonable, but noisy, fit.

Following the above reasoning, thus, we found that the stretched exponential distribution most often fitted the sleep bout duration distributions of the 6–10 days olds (*k* = 0.69 ± 0.03, λ = 20 ± 7 s), 4–6 weeks (*k* = 0.77 ± 0.03, λ = 16 ± 2 s), and 4–6 months (*k* = 0.71 ± 0.02, λ = 14 ± 2 s). No statistically significant difference on the mean values was found between the shapes *k* of the different age groups (**Figure 6E**) or of the scales λ. **Figures 6F,G** depict sleep bout and wake bout distributions, respectively. For the 12+ month old group the power-law and the lognormal distributions tied with equal number of consistent cases, however, applying AICc on the two cases where both models were plausible tipped the scale in favor of the power-law distribution (α = 2.8 ± 0.2). For all age groups we thus observe a sleep distribution indicative of bursty dynamics, where longer sleep bouts occur more often than in the random (exponential distribution) case. For the wake bout distributions the power-law was favored at all ages, with α = 4.1 ± 0.4 for 6- to 10-day-olds, α = 4.0 ± 0.1 for the 4- to 6-week group, α = 2.6 ± 0.1 for the 4–6 months, and α = 2.4 ± 0.1 for the zebrafish older than 12 months of age and a decrease of the power-law exponent α was seen with age [*F*(3,57) = 12.1, *p* < 0.0001]. This is indicative of a strongly bursty dynamics where once an awakening has occurred, the probability of falling back to sleep again shortly, decreases with age. The wake bout distributions of adult fish (4–6 months and 12+ months) had significantly lower exponents than the wake bout distributions of the larval stages (6–10 days and 4–6 weeks), *p* < 0.0001, **Figure 6H**. In concordance with previous study by Prober et al. (2006) sleep and wake bouts in larval zebrafish were further analyzed using a 60-s immobility threshold for sleep onset. As expected, sleep percentage decreases whereas wake percentage increases using this criteria (20.3 and 79.7%, respectively). Full list of sleep parameters using a 60-s threshold are depicted in **Table 2**. Sleep and wake bouts, however, maintain their respective distributions: we found that the stretched exponential distribution most often fitted the sleep bout duration distributions (*k* = 0.928 ± 0.049, λ = 73.234) and the power-law most often fitted the wake bouts (α = 2.651 ± 0.234).

**Table 2:** Sleep and wake parameters in larval zebrafish using a 60 immobility threshold.

	Threshold (s)	Percent time of night (%)		Bout length (min)		Total number of bouts		Fragmentation (bouts/min)		Number of different bout lengths	
		Mean	SE	Mean	SE	Mean	SE	Mean	SE	Mean	SE
Sleep	60	20.3	6.6	1.3	0.3	73.0	15.7	1.18	0.166	47.2	8.8
Wake	60	79.7	6.6	12.1	2.6	72.8	15.7	0.23	0.067	42.1	7.1

## DISCUSSION

We characterize the ontogeny of sleep–wake cycles in zebrafish and by defining sleep architecture in terms of state transitions, we demonstrate that sleep–wake cycles in zebrafish develop in a trajectory that can be meaningfully compared to humans (Kleitman and Engelman, 1951; Roffwarg et al., 1966; Jenni et al., 2006). By showing that sleep architecture in humans and zebrafish can be directly contrasted using multiple measures, zebrafish are further validated as a highly useful sleep model (Rihel et al., 2010b).The similarities demonstrated between zebrafish (order: Cyprinidae) and humans is consistent with the notion of evolutionarily conserved neural substrates controlling the sleep states.

In the present study, we replicate and extend previous findings on human sleep development. We show that sleep percentage overnight decreases with age whereas sleep bout length, sleep bout number and sleep fragmentation do not change with age. Since the recordings are done during night only, analyzing the wake bouts (the interruption of sleep) is rich with information on sleep quality. We show that wake time increases with age and so does average wake bout length. There is no change in the wake bout number but wake fragmentation does decrease with age. In short, sleep percentage decreases with age and the decrease is explained by longer wake bouts and not by an increase in the number of wake bouts during night. It should be stressed that these data reflect only nighttime recordings and the youngest participant was 2 years old. Generally, across a full 24-h period, sleep fragmentation decreases rapidly over the first year in humans (Kleitman and Engelman, 1951; Jenni et al., 2006).

Second, in zebrafish we show that sleep percentage also decreases with age. Similar to humans, average sleep bout length and sleep fragmentation did not change with age, however, in contrast to humans, sleep bout number also decreased. The drop in sleep percentage in zebrafish with age is due to fewer but not shorter sleep bouts. We show that, similar to humans, wake percentage increases, the average wake bouts increase in length and wake fragmentation decreases (i.e., once awake the probability of falling asleep again decreases) with age. In contrast to humans, wake bout number decreases with age in zebrafish. Thus, in terms of these four parameters, percentage, bout length, bout number and fragmentation, zebrafish and humans follow a highly similar developmental trajectory. They differ only in the change of the number of sleep–wake transitions with age (no change in humans but a decrease in zebrafish).

Third, consistent with recent findings (Chu-Shore et al., 2010) we reveal that sleep–wake bout length distributions are more complex than suggested by previous work. Previously it has been shown that wake bouts exhibit a scale-free power-law distribution with an exponent that remains constant across adult humans, cats, rats, and mice (Lo et al., 2002, 2004). In contrast, it was also shown that sleep bout durations follow an exponential distribution with a characteristic time scale whose main determinants are body size and metabolic rate (Lo et al., 2002, 2004). Turning to development, it was shown in neonatal rats and mice that both sleep and wake bouts exhibit an exponential distribution immediately after birth, with a power-law behavior of wake bouts emerging only after the second postnatal week (Blumberg et al., 2005, 2007a, 2b). A similar developmental trajectory of sleep–wake dynamics has been reported in sheep (Karlsson et al., 2011). The data from all these species, therefore, indicated that the power-law exponent, α, is constant across multiple adult species; in contrast, the sleep-related time constant τ varies across species and age (Lo et al., 2002, 2004). Previous studies have, however, reported age-restricted exponential behavior of sleep bout distributions (Arnardóttir et al., 2010). Regardless, the conformity of the data from adult cats, rats, mice and humans (Lo et al., 2002, 2004) as well as from developing rats and mice (Blumberg et al., 2005, 2007a), supported the notion that these trends in sleep–wake bout distribution represent universal mammalian phenomena. It is tempting to speculate that similar bout distributions can be measured in invertebrates such as *Drosophila*. Presently we show that of the models tested, sleep bouts are most consistent with a stretched exponential in humans – except for in the 50+ group were they exhibit a better fit to a power-law. Wake bouts were shown to have a better fit to a power-law at all ages. Humans under the age of two might exhibit different distributions, even though wake bouts in prematurely born humans do not show a better fit to either a power-law or an exponential distribution (Arnardóttir et al., 2010). Similarly, in zebrafish sleep bout distributions most often fitted with a stretched exponential but in the 12+ month old group the power-law distribution showed better fits. Also, we demonstrate that α decreases with age in zebrafish; α is thus not a stable species characteristic in zebrafish, but the adult values compare well to those of mammals.

Brief awakenings during night are not random disruptions of sleep but a regulated process that may reveal the underlying mechanisms of behavioral state control (Lo et al., 2002, 2004; Bianchi et al., 2010; Chu-Shore et al., 2010). Previous rodent work has shown that the consolidation of wake bouts, and the concurrent emergence of a power-law wake bout distribution, across development, depends on intact hypothalamic-to-brainstem connections (Karlsson et al., 2004). This developmental trend may depend on hypocretin neurons, whose caudal connections develop in concert with the emergence of the power-law; since both hypocretin knock-out or locus coeruleus lesions (containing the highest density of hypocretin receptors) reverse the trend (Blumberg et al., 2007a; Gall et al., 2009). It is parsimonious to assume that the same underlying neural circuitry explains the bout length distributions in zebrafish as in mammals. Gall et al. (2009) stress that scale-free networks are more robust and resistant to failure than are random networks (Albert et al., 2000) and suggest that this organization evolved to protect the waking state from random neural damage. At face value this idea seems plausible since an organism partly awake cannot forage, mate, etc. Moreover, it follows that since the sleep state is organized in a less robust manner it should be more prone to failures – and sleep disorders are among the most common disorders (Partinen and Hublin, 2005). This notion does no account for the stretched exponential and the emergence of a power-law behavior of sleep bouts in either older zebrafish or humans, however, which implies that the sleep state has more structure than was previously thought. Regardless, the fact that both species exhibit similar organization in sleep architecture, i.e., both species exhibit transition from stretched exponential to power-law behavior of the sleep bouts at the oldest age tested, is consistent with the notion that there are conserved evolutionary constraints on the structure of neural circuits governing sleep and wake.

Even though we have the utmost confidence in our data and analysis methods, some potential drawbacks of our study should be discussed. The first is the choice of age groups. Immense efforts have been made to generate tools to meaningfully compare different model species across development (see ; Clancy et al., 2001). Unfortunately, no such efforts have been for made for zebrafish making the choice of age groups difficult. The choice of age groups, both for humans and zebrafish, is meant to capture general trends in sleep development across the lifespan. Judging from the rapid fall in sleep percentages between 6–10 days and 4–6 weeks it seems likely that the largest differences in sleep development are to be found over the first weeks post-fertilization in zebrafish. In mammals, the largest changes also take place at a very early age (Jouvet-Mounier et al., 1970; Blumberg et al., 2005; Jenni et al., 2006). A detailed comparison of sleep–wake cycle development in zebrafish with dense group sampling over the first weeks post-fertilization may be warranted. This is also a period of synaptogenesis and pruning of neural circuits and also the age in which the fish are, conveniently, highly amenable to many of the molecular and neurophysiological tools available (Fetcho, 2005, 2007; McLean and Fetcho, 2008; Appelbaum et al., 2010; Friedrich et al., 2010; Vargas et al., 2012). We suggest that in terms of delineating the neural circuits of sleep, focusing on mapping behavioral to neural changes over this period in early ontogeny will be fruitful. Another valid critique is our choice of temperature. Total sleep time varies with basal metabolic rate (Zepelin and Rechtschaffen, 1974) which in turn is affected by temperature (Clarke and Johnston, 1999). It is conceivable that different values for total sleep time, or other sleep parameters, would be attained when recording at different temperature. In the current experiment we restricted ourselves to temperatures used by other authors (Zhdanova et al., 2006; Yokogawa et al., 2007; Appelbaum et al., 2009; Rihel et al., 2010b). Lastly, we use 6 s of immobility as our threshold for sleep onset for all our age groups. This was the immobility-to-sleep threshold measured by Yokogawa et al. (2007) in adult zebrafish. In that study the authors used mild electric shock to first find a threshold for arousal. Next, they applied the same stimulus to zebrafish, at different time points after the onset of immobility, determining that this arousal threshold rose after 6 s. That is, after 6 s, on average, larger stimulus was needed to generate a response. While choosing to use the low value (6 s) for all the age groups we do not feel that the 60-s threshold is inadequate; larval fish, however, exhibit shorter average wake (i.e., mobility) bouts than older fish, while average sleep bout lengths remain constant across age. This is evidence of a more rapid average sleep onset in larvae and is consistent with the use of the same immobility time threshold for sleep in young and old fish. Analyzing the bout distribution data using a 60-s immobility threshold for sleep onset reveals that sleep parameters are altered. Sleep percentage and number of bouts are reduced whereas the basic structure of bout distributions is conserved. For direct comparison between zebrafish sleep studies all parameters, e.g., movements and time thresholds need to be standardized and made fully comparable. To this end, more studies are needed.

The method of classifying sleep–wake states used here only requires knowledge on the duration of sleep and wake bouts, as opposed to the detailed knowledge of REM–NREM alternations, micro-arousals, or other phenomena required for traditional analysis methods in mammals (Rechtschaffen and Kales, 1968). We have shown that this method is highly sensitive to developmental changes and therefore could be used for gaging developmental milestones. Since electrographic criteria are not necessary for the analysis, data from a large group of animals that have only recently begun to be used in sleep research, such as fruit flies, zebrafish, and even nematodes (Hendricks et al., 2000; Shaw et al., 2000; Zhdanova, 2006; Zhdanova et al., 2006; Raizen et al., 2008), can be analyzed in a manner directly and meaningfully comparable to humans. These findings argue for evolutionary conserved neural substrates controlling sleep and further solidify zebrafish as a valuable model in sleep research.

## Conflict of Interest Statement

The authors declare that the research was conducted in the absence of any commercial or financial relationships that could be construed as a potential conflict of interest.
